# Dual-emissive nanoarchitecture of lanthanide-complex-modified silica particles for *in vivo* ratiometric time-gated luminescence imaging of hypochlorous acid†
†Electronic supplementary information (ESI) available: Characterization of the new compounds, Fig. S1–S12 and a video showing the real-time uptake and accumulation of HClO in living *Daphnia magna* using the RTLNP as a probe. See DOI: 10.1039/c6sc02243j



**DOI:** 10.1039/c6sc02243j

**Published:** 2016-07-29

**Authors:** Hua Ma, Bo Song, Yuanxiu Wang, Deyuan Cong, Yufei Jiang, Jingli Yuan

**Affiliations:** a State Key Laboratory of Fine Chemicals , School of Chemistry , Dalian University of Technology , Dalian 116024 , P. R. China . Email: bo.song@dlut.edu.cn ; Email: jlyuan@dlut.edu.cn ; Fax: +86-411-84986042

## Abstract

We have developed a ratiometric time-gated luminescence sensory system for *in vivo* imaging of hypochlorous acid (HClO) by preparing a dual-emissive nanoarchitecture of europium- and terbium-complex-modified silica nanoparticles. The design of this nanoarchitecture is based on our new finding that the strong, long-lived luminescence of the β-diketonate–Eu^3+^ complex can be rapidly and selectively quenched by HClO. Therefore, the β-diketonate–Eu^3+^ complex was decorated on the surface of the silica nanoparticles for responding to HClO, while a HClO-insensitive luminescent terbium complex was immobilized in the inner solid core of the nanoparticles to serve as an internal standard. This nanosensing probe combines the advantages of both ratiometric and time-gated detection modes to afford high accuracy and sensitivity. Upon exposure to HClO, the nanoprobe displayed a remarkable luminescence color change from red to green, and the intensity ratio of the green over the red luminescence (*I*_539_/*I*_607_) showed a rapid, sensitive and selective response to HClO. Additionally, the feasibility of using the nanoprobe for intracellular detection of exogenous and endogenous HClO and for real-time mapping of HClO in small laboratory animals has been demonstrated *via* ratiometric time-gated luminescence imaging microscopy. The results reveal that the constructed nanoarchitecture cloud is a favorable and useful sensing probe for the real-time imaging of HClO *in vivo* with high specificity and contrast.

## Abstract 2

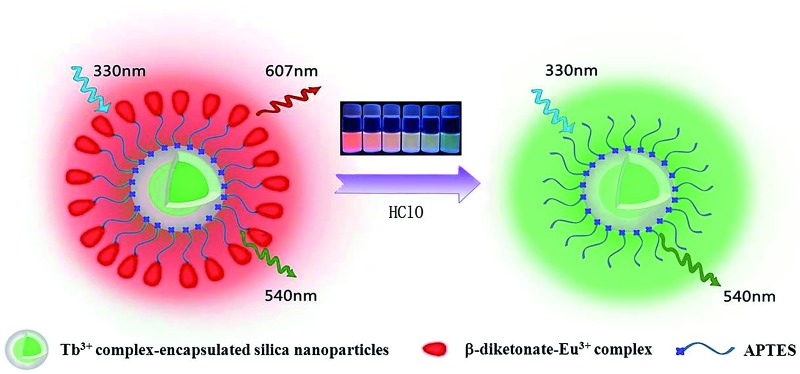
A dual-emissive nanoarchitecture of lanthanide-complex-modified silica particles was developed for real-time ratiometric time-gated luminescence imaging of HClO *in vivo*.

## Introduction

Unlike most other reactive oxygen species (ROS) and reactive nitrogen species (RNS), hypochlorite (ClO^–^) and its protonated form, hypochlorous acid (HClO), are widely employed in our daily lives as bactericides and bleaching agents.^1^ In biological systems, ClO^–^, which is endogenously produced from the reaction of a chloride ion and hydrogen peroxide catalyzed by the enzyme myeloperoxidase (MPO) in leukocytes,^2^ plays a vital role in killing a wide range of pathogens.^3^ However, ClO^–^ may also cause extensive oxidative stress and damage to nucleic acids, proteins and lipids,^4,5^ and there is evidence that misregulated HClO contributes to the tissue injury associated with inflammation.^6^ An increasing number of studies have revealed that neutrophil-derived HClO was related to hepatic ischemia-reperfusion injury,^7^ rheumatiod arthritis,^8^ lung injury,^9^ atherosclerosis,^10^ and renal disease.^11^ Nevertheless, the biological activity of HClO has not yet been fully revealed. To fully understand the physiological and pathological roles of HClO, highly sensitive and selective monitoring and imaging of HClO in living systems are vitally important and necessary.

**Fig. 1:**
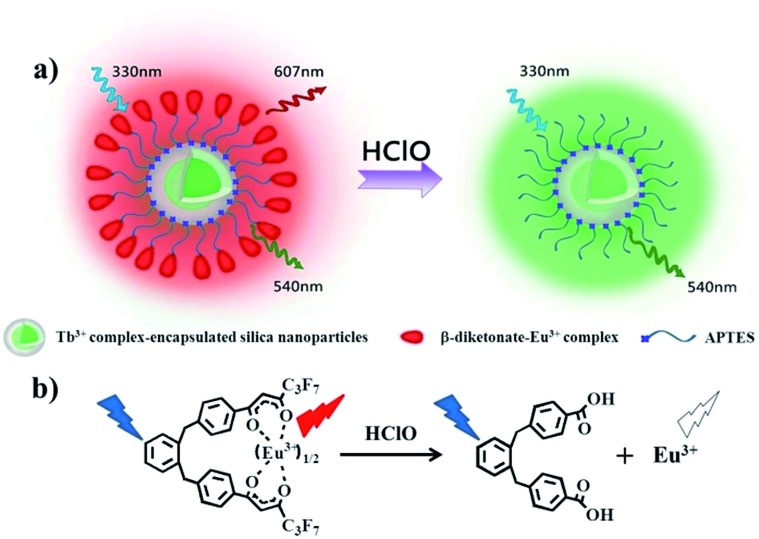
Design concept of a ratiometric luminescence probe based on crown-like dual-emissive silica nanoparticles modified by Tb^3+^ and Eu^3+^ complexes (a), and the luminescence quenching mechanism of a β-diketonate–Eu^3+^ complex (BHHBB–Eu^3+^) by HClO (b).

Various analytical methods for detecting HClO have been developed, including electrochemical,^12^ chemiluminescence,^13^ colorimetric^14^ and fluorescence^15^ methods. Among them, the fluorescence imaging method is normally superior in terms of its high sensitivity, spatiotemporal resolution, and operational simplicity. A number of optical sensing probes including organic dyes,^16–19^ transition metal complexes,^20–22^ lanthanide complexes^23^ and fluorescence nanoparticles^24–26^ have been developed to detect HClO in living cells, tissues and laboratory animals. However most of them are intensity-based fluorescent probes, and a variety of analyte-independent factors, such as instrumental parameters, the microenvironment around the probe molecules, probe distribution and photobleaching, can interfere with the signal output. It is well known that ratiometric fluorescent probes are more reliable than intensity-based probes because they can normalize these interferences by the built-in correction of two emission bands,^27^ but unfortunately the use of ratiometric probes is relatively rare in contrast to intensity-based probes because of the complexity of the design and synthesis of ratiometric probes. Here, crown-like dual-emissive silica nanoparticles were chosen to construct a ratiometric luminescence probe because of their excellent hydrophilicity, biocompatibility, stability and convenient modification to meet all sorts of requirements.^28^ As shown in Fig. 1, the nanoparticle's core is covalently doped with a reference dye which is inert to the analyte to serve as an internal standard, while an analyte-responsive compound is covalently linked on the surface of dye-encapsulated silica nanoparticles to form the outer crown. Upon interaction with the analyte, the emissions of the nanoparticles will display a ratiometric luminescence response to the analyte.

Under physiological conditions, HClO is highly reactive and short-lived,^29^ so a probe with a fast response and high selectivity and sensitivity is desirable for real-time monitoring of HClO in biological samples. On the other hand, a lot of studies have shown that the time-gated luminescence technique using lanthanide complexes as probes is a more efficient approach to enhancing the sensing sensitivity of the probes. In contrast to conventional organic fluorescence probes, luminescent lanthanide (mainly Eu^3+^ and Tb^3+^) complexes possess super long-lived luminescence with large Stokes shifts and sharp emission profiles, which permits the use of the time-gated detection mode to eliminate the interference of autofluorescence and scattering lights, thus remarkably improving the signal-to-noise contrast ratio and sensitivity.^30–34^

In this work, we report for the first time that β-diketonate–Eu^3+^ complexes show a sensitive, selective and rapid turn-off luminescence signal in response to HClO, and can serve as HClO-responsive luminophores. Furthermore another luminescent Tb^3+^ complex that is insensitive to HClO can be employed as a reference dye. When the surface of the Tb^3+^ complex-encapsulated silica nanoparticles is decorated with β-diketonate–Eu^3+^ complex molecules, a ratiometric time-gated luminescence nanoprobe (RTLNP) for HClO is constructed. This RTLNP combines both the advantages of ratiometric and time-gated detection modes to afford high accuracy and sensitivity. The utility of the constructed nanoprobe, the RTLNP, was demonstrated by the ratiometric time-gated luminescence detection of HClO in aqueous media, living cells and laboratory animals, respectively.

## Results and discussion

### Selection of the responsive and reference lanthanide complexes for construction of the RTLNP

**Fig. 2:**
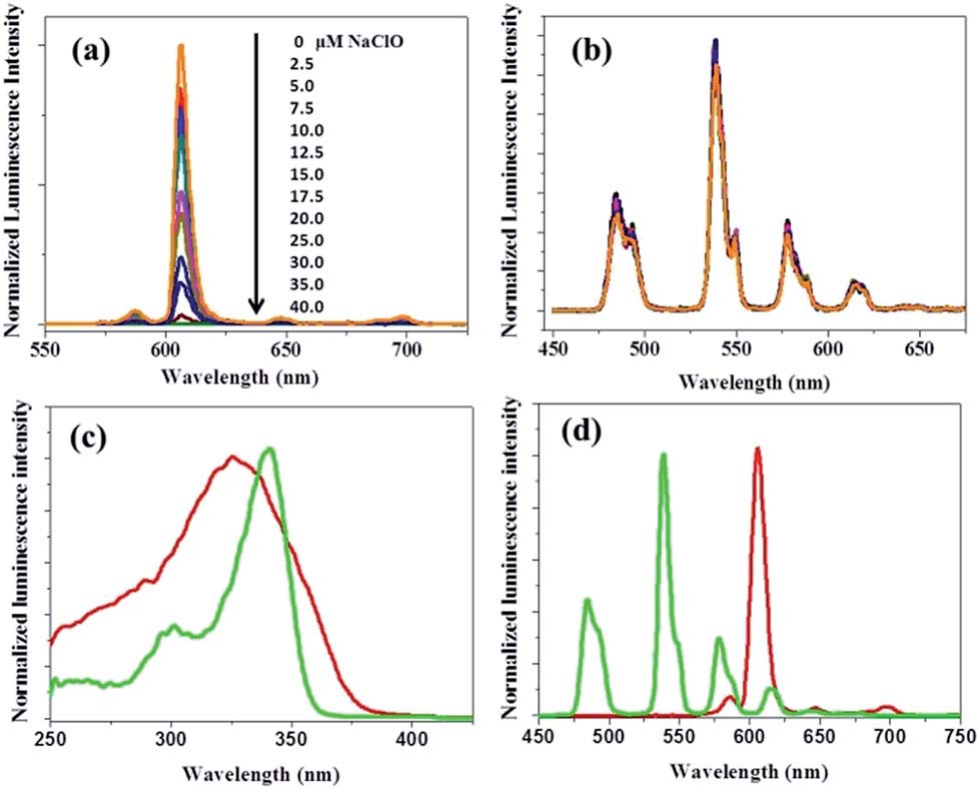
(a) Time-gated emission spectra of BHHBB–Eu^3+^ (5.0 μM) in 1 : 4 ethanol–0.05 M PBS buffer of pH 7.4 in the presence of 0–40 μM NaClO. (b) Time-gated emission spectra of PTTA–Tb^3+^ (20 μM) in 1 : 4 ethanol–0.05 M PBS buffer of pH 7.4 in the presence of 0–40 μM NaClO. (c) Time-gated excitation spectra of BHHBB–Eu^3+^ (red) and PTTA–Tb^3+^ (green) in 1 : 4 ethanol–0.05 M PBS buffer of pH 7.4. (d) Time-gated emission spectra of BHHBB–Eu^3+^ (red) and PTTA–Tb^3+^ (green) in 1 : 4 ethanol–0.05 M PBS buffer of pH 7.4.

For construction of the RTLNP, the first step was to select the reference and responsive lanthanide luminophores. A notable finding in this work was the high responsiveness of the β-diketonate–Eu^3+^ complex to HClO. We find for the first time that the luminescence of β-diketonate–Eu^3+^ complexes can be rapidly, selectively and sensitively quenched in the presence of HClO. Thus, we chose a newly developed β-diketonate–Eu^3+^ complex, 1,2-bis[4′-(1′′,1′′,1′′,2′′,2′′,3′′,3′′-heptafluoro-4′′,6′′-hexanedion-6′′-yl)-benzyl]-4-benzene–Eu^3+^ (BHHBB–Eu^3+^), as a HClO-responsive lanthanide luminophore because of its excellent properties of strong luminescence and long emission lifetime.^35^ Furthermore, BHHBB–Eu^3+^ is easy to functionalize (such as by chlorosulfonation) and covalently conjugate to silica nanoparticles.^35,36^ As shown in Fig. 2a, upon reaction with different concentrations of HClO, the long-lived red luminescence of BHHBB–Eu^3+^ gradually decreased until full disappearance, which enables the ratiometric measurement to be high contrast. To elucidate the mechanism of the HClO-mediated luminescence quenching of BHHBB–Eu^3+^, BHHBB–Eu^3+^ and HClO were mixed in a 1 : 1 acetonitrile–0.05 M PBS buffer (pH 7.4), and the product, purified by silica gel column chromatography, was characterized by ESI-MS, ^1^H NMR and ^13^C NMR (Fig. S10–S12 in the ESI†). The results show that the carbonyl group of β-diketonate was completely oxidized by HClO to generate a carboxylic acid (Fig. 1), which leads to the decomposition and luminescence quenching of BHHBB–Eu^3+^.

With a red luminescent Eu^3+^ complex that can respond to HClO, a green luminescent Tb^3+^ complex should be selected as a reference dye. Here, a strongly luminescent Tb^3+^ complex, *N*,*N*,*N*′,*N*′-(4′-phenyl-2,2′:6′,2′′-terpyridine-6,6′′-diyl) bis(methylenenitrilo) tetraacetate–Tb^3+^ (PTTA–Tb^3+^, Fig. S1†), was chosen as a reference dye because it is highly stable and does not show any notable changes in its time-gated luminescence spectrum upon exposure to HClO (Fig. 2b). It is noteworthy that the maximum excitation wavelengths of BHHBB–Eu^3+^ and PTTA–Tb^3+^ are significantly overlapped (Fig. 2c) and their respective maximum emission wavelengths are spectrally well-separated (Fig. 2d). Additionally, both complexes retained their luminescence intensities when mixed with each other, indicating limited energy, lanthanide ion or electron transfer between two complexes. All of these photophysical properties and sensory behaviors of the selected lanthanide complexes fulfilled the requirements of cooperation in a hybrid ratiometric time-gated luminescent sensory system.

### Preparation of the RTLNP for HClO detection

**Fig. 3:**
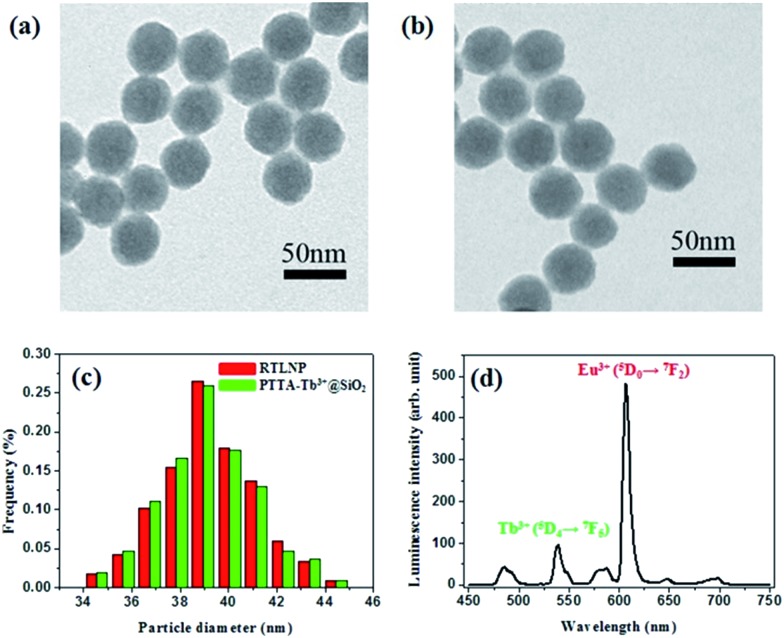
TEM images of PTTA–Tb^3+^-encapsulated silica particles (a) and RTLNP particles (b). (c) Particle size distribution histograms of the two kinds of nanoparticles. (d) Time-gated emission spectrum of the RTLNP (50 μg ml^–1^) in 1 : 4 ethanol–0.05 M PBS buffer of pH 7.4 (*λ*_ex_ = 330 nm).

The key element for preparing the RTLNP is to decorate BHHBB–Eu^3+^ molecules onto the surface of silica nanoparticles. In the previously reported approach for building a core–shell structured ratiometric fluorescent nanoprobe, the reference dye-encapsulated silica nanoparticle was coated with a mesoporous silica layer, and then the responsive dye was decorated into the outer shell by physical absorption.^37^ However this nanoprobe might suffer from leakage of absorbed dye molecules, which could interfere with the signal stability of the nanoprobe. In this work, we firstly conjugated the PTTA–Tb^3+^ and chlorosulfonated BHHBB–Eu^3+^ to (3-aminopropyl) triethoxysilane (APTES). After two silylated dye derivatives, APTES–PTTA–Tb^3+^ and APTES–BHHBB–Eu^3+^, were obtained, the PTTA–Tb^3+^-doped silica cores (PTTA–Tb^3+^@SiO_2_) were prepared by the hydrolysis and copolymerization of APTES–PTTA–Tb^3+^ and tetraethyl orthosilicate (TEOS) in a W/O microemulsion according to our previous method.^38,39^ Then the PTTA–Tb^3+^@SiO_2_ nanoparticles were covalently incorporated into APTES–BHHBB–Eu^3+^, which enables the dye leakage to be minimized effectively.^40^ The transmission electron microscopy (TEM) and scanning electronic microscopy (SEM) images showed the spherical structure, high dispersity and good homogeneity of the as-prepared nanoparticles (Fig. 3a and b and S2†). Two kinds of nanoparticles, PTTA–Tb^3+^@SiO_2_ particles and BHHBB–Eu^3+^-decorated particles (the RTLNP), were well dispersed in the solution (Fig. 3a and b) with mean diameters (±SD) of 39 ± 5 nm and 40 ± 5 nm, respectively, which indicates that the covalent immobilization of BHHBB–Eu^3+^ on the surface of the PTTA–Tb^3+^@SiO_2_ nanoparticles did not significantly change the sizes of the particles (Fig. 3c). Furthermore, no dark dots were observed in the silica sphere, which implies that the covalently bound PTTA–Tb^3+^ molecules are well distributed in the particles. Moreover, dynamic light scattering (DLS) and zeta potential measurements were performed using a Nano-Zetasizer. The DLS analysis (Fig. S3†) of the RTLNP shows an average hydrated particle diameter of 241.4 nm, which is significantly higher than the diameter of an individual RTLNP (40 nm) evaluated by TEM images and suggests that the RTLNP particles can be dispersed in water, but may be in the form of small aggregates. As shown in Fig. 3d, the time-gated emission spectrum of the RTLNP exhibited the typical Tb^3+^ and Eu^3+^ emissions consisting of several discrete bands between 455 and 710 nm corresponding to the ^5^D_4_ → ^7^F_*J*_ (*J* = 6–3) transitions of Tb^3+^ and the ^5^D_0_ → ^7^F_*J*_ (*J* = 0–4) transitions of Eu^3+^. The luminescence lifetimes of PTTA–Tb^3+^ and BHHBB–Eu^3+^ immobilized in the RTLNP were measured to be 1.28 ms and 0.60 ms, respectively. Since PTTA–Tb^3+^ was effectively protected from luminescence quenching by the silica matrix, the luminescence lifetime of PTTA–Tb^3+^ encapsulated in silica nanoparticles is much longer than that of free PTTA–Tb^3+^ (0.45 ms).^41^ However, because BHHBB–Eu^3+^ was decorated on the surface of nanoparticles, the luminescence lifetime of BHHBB–Eu^3+^ in the RTLNP is similar to that of free BHHBB–Eu^3+^ (0.53 ms in 1 : 4 ethanol–0.05 M PBS buffer of pH 7.4). Additionally, the luminescence spectrum of the RTLNP in aqueous buffers (even containing 25% ethanol or 5% Triton X-100) did not show any remarkable changes when the solution was left to stand for more than four weeks. The loading amounts of lanthanide complexes as measured by inductively coupled plasma-optical emission spectroscopy (ICP-OES) were calculated to be about 29 nmol BHHBB–Eu^3+^ and 25 nmol PTTA–Tb^3+^ per 1.0 mg of nanoparticles. These results clearly indicate that two kinds of lanthanide luminophores have been covalently immobilized in the silica nanoparticles, and as-prepared RTLNP particles were suitable for use in the time-gated luminescence measurements.

### Luminescence response of the RTLNP to HClO

**Fig. 4:**
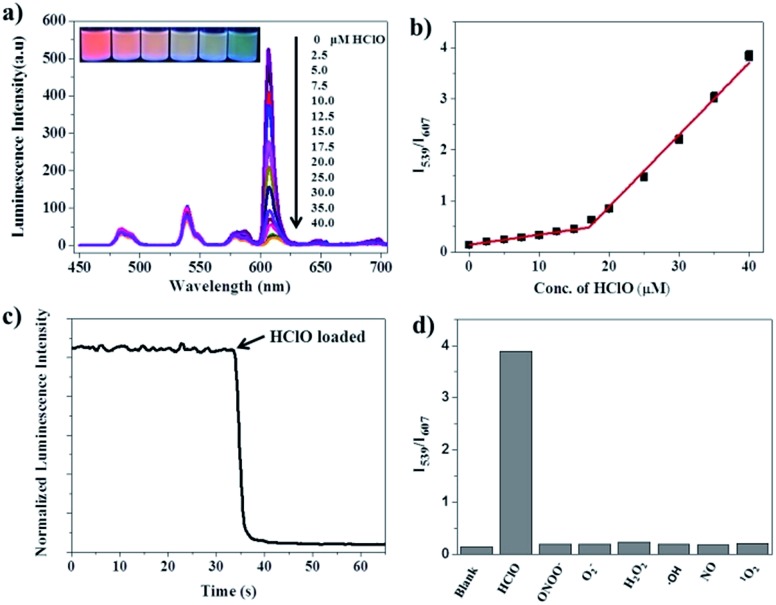
(a) Time-gated emission spectra (*λ*_ex_ = 330 nm) of the RTLNP (50 μg ml^–1^) in the presence of different concentrations of HClO in 1 : 4 ethanol–0.05 M PBS buffer of pH 7.4 (the inset shows the luminescence colors of the solutions under a 360 nm UV lamp). (b) The *I*_539_/*I*_607_ ratio response of the RTLNP to different concentrations of HClO. (c) Time course of luminescence intensity of the RTLNP at 607 nm upon addition of 100 μM HClO. (d) The *I*_539_/*I*_607_ ratio response of the RTLNP (50 μg ml^–1^) toward HClO (40 μM) and other ROS and RNS (100 μM).

The luminescence response of the RTLNP to HClO was investigated in 1 : 4 ethanol–0.05 M PBS buffer of pH 7.4. In the absence of HClO, the RTLNP displayed a Eu^3+^–Tb^3+^ dual-emission spectrum with two well-resolved main emission peaks at 539 nm (^5^D_4_ → ^7^F_5_ transition of Tb^3+^) and 607 nm (^5^D_0_ → ^7^F_2_ transition of Eu^3+^) under excitation at the same wavelength (330 nm). Upon addition of HClO, the luminescence of the RTLNP at 607 nm exhibited substantial quenching, while that at 539 nm was only slightly changed, which is in line with the luminescence responses of BHHBB–Eu^3+^ and PTTA–Tb^3+^ toward HClO. Moreover, the red luminescence of the RTLNP gradually decreased upon increasing the HClO concentration, accompanied by distinguishable luminescence color changes from red to green (the inset in Fig. 4a). The intensity ratio of the green over the red luminescence (*I*_539_/*I*_607_) was increased from 0.14 to 3.84 upon increasing the HClO concentration from 0 μM to 40 μM, which provided a 27-fold contrast window for the detection of HClO. Furthermore, the dose-dependent *I*_539_/*I*_607_ ratio changes of the RTLNP showed a complicated behavior with two linear dynamic ranges against the increase of HClO concentration (Fig. 4b). This phenomenon might be caused by the two-step reaction of HClO with two β-diketonate ligands of the BHHBB–Eu^3+^ complex. The detection limit for HClO, calculated as the concentration corresponding to triple standard deviations of the background signal,^36^ is 0.27 μM. The dynamic range covers the physiological concentration range of HClO (5–25 μM),^24^ suggesting that the RTLNP could act as an ideal ratiometric time-gated luminescence probe for the quantification of HClO in biological systems.

Since a short response time is essential to the real-time detection of HClO under physiological conditions, the time-dependent luminescence decrease (at 607 nm) of the RTLNP in the presence of HClO was investigated. As shown in Fig. 4c, upon addition of HClO, the red luminescence of the RTLNP was rapidly quenched and reached a steady value within 5 seconds. This result indicates that the RTLNP can quickly respond to HClO, which is beneficial for the detection of short-lived HClO. In addition, the reactions of the RTLNP with ROS and RNS that are commonly present in biological matrices were examined under the same conditions to evaluate the response specificity of the RTLNP to HClO. As shown in Fig. 4d, the ratiometric time-gated luminescence response was highly specific toward HClO over other ROS and RNS including H_2_O_2_, O_2_^–^˙, ˙OH, ^1^O_2_, ONOO^–^ and NO. All of the above results demonstrate that the RTLNP particles hold promise as sensitive and selective nanoprobes for the detection of HClO, which enables the real-time generation of HClO in complicated biosystems to be monitored without significant interferences of other biological and reactive species.

### Application of the RTLNP for ratiometric time-gated luminescence imaging of intracellular HClO

**Fig. 5:**
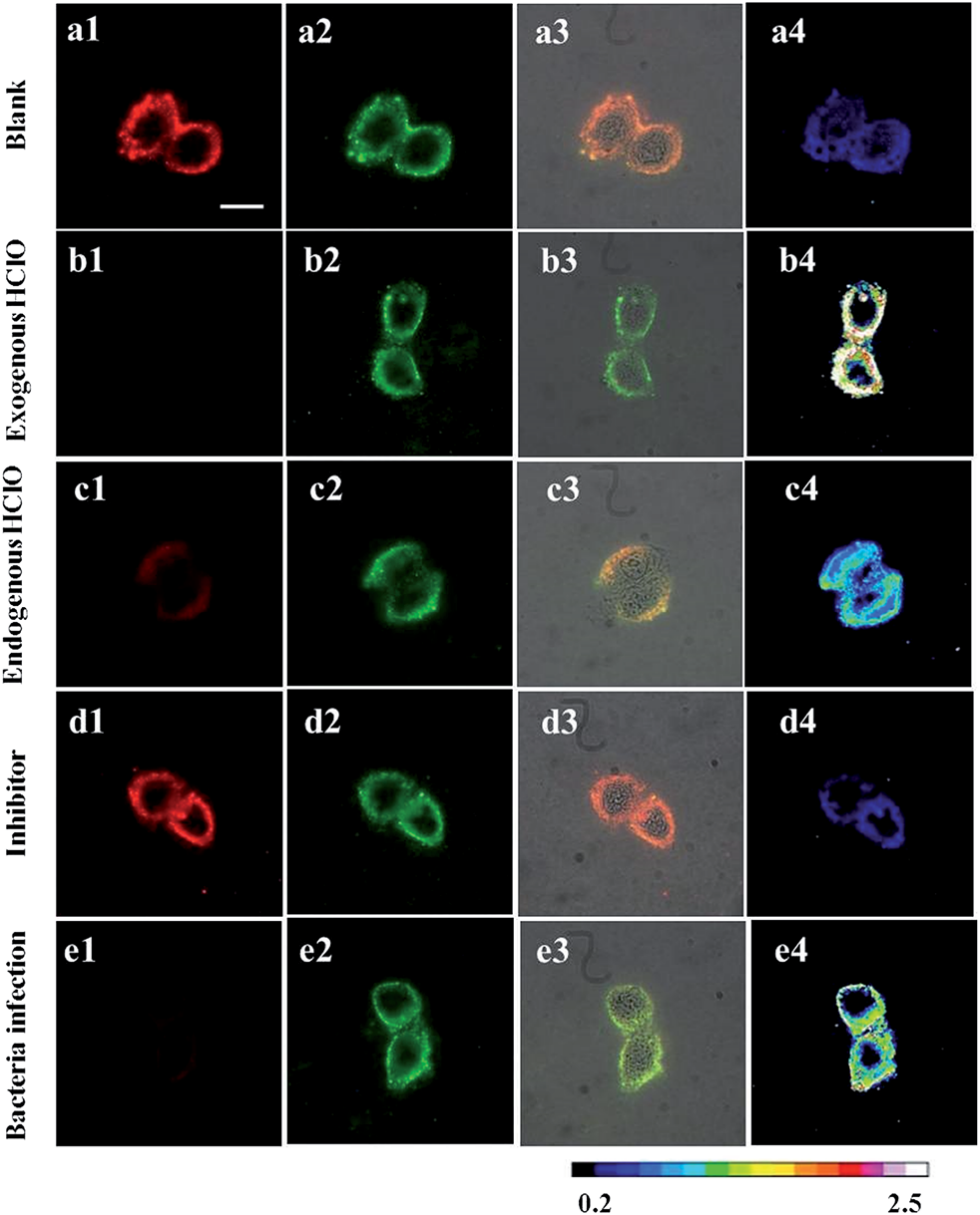
Time-gated luminescence images of the RAW 264.7 cells (1: the images at the red channel; 2: the images at the green channel; 3: overlap of luminescence and bright field images; 4: *I*_green_/*I*_red_ ratiometric images. Excitation filter: 330–380 nm. Scale bar: 20 μm). (a) The RTLNP-loaded cells before treatment with HClO. (b) The RTLNP-loaded cells were incubated with HClO (50 μM) for 10 min. (c) RAW 264.7 cells were incubated with LPS (100 ng ml^–1^) for 16 h, followed by incubation with IFN-γ (50 ng ml^–1^) for 4 h, PMA (10 nM) for 30 min, and 50 μg ml^–1^ RTLNP for 1.5 h, respectively. (d) RAW 264.7 cells were incubated with LPS (100 ng ml^–1^) for 16 h, IFN-γ (50 ng ml^–1^) for 4 h, PMA (10 nM) and 4-ABAH (50 μM) for 2 h, and 50 μg ml^–1^ RTLNP for 1.5 h, respectively. (e) RAW 264.7 cells were pretreated with *E. coli* (2 × 10^5^ CFU ml^–1^) for 7 h, followed by incubation with 50 μg ml^–1^ RTLNP for 1.5 h.

Before investigating the intracellular sensing behavior of the RTLNP toward HClO, the influence of the RTLNP on cell proliferation and viability was examined using the 3-(4,5-dimethyl-2-thiazolyl)-2,5-diphenyl-2-*H*-tetrazolium bromide (MTT) assay method.^42^ As shown in Fig. S5,† the cell viabilities remained still at above 90% after incubation with different concentrations of the RTLNP (up to 120 μg ml^–1^) for 24 h, which indicates that the RTLNP particles are biocompatible with low cytotoxicity for the luminescent sensing of HClO in living cells. Based on this result, the ratiometric time-gated luminescence response of the RTLNP to intracellular HClO in living RAW 264.7 cells was investigated. For proof-of-concept, the RTLNP was initially used to image the exogenous HClO in the RAW 264.7 cells. The RAW 264.7 cells were incubated with 50 μg ml^–1^ RTLNP in an isotonic saline solution containing 1.0 mg ml^–1^ solubilizer, cremophor C040, for 1.5 h at 37 °C, and then the RTLNP-loaded cells were treated with a blank control or HClO (50 μM) for another 10 min under the same conditions. The luminescence images of the cells were recorded on a true-color time-gated luminescence microscope.^43^ Before HClO treatment, it was clearly observed that both the green (540 ± 25 nm) and red (>590 nm) channels exhibited bright time-gated luminescence signals in the cytoplasm of the RTLNP-loaded cells (Fig. 5a). After the RTLNP-loaded cells were incubated with HClO, a remarkable decrease of red intracellular luminescence was observed while the green intracellular luminescence was not obviously changed (Fig. 5b). The intensity ratio of the green emission over the red one, *I*_green_/*I*_red_, was determined to increase from 0.21 (Fig. 5a4) to 2.25 (Fig. 5b4) upon the HClO-induced oxidative decomposition of BHHBB–Eu^3+^ on the surface of the RTLNP, which is consistent with the results of the luminescence titration in the buffer, and indicates that the RTLNP can be easily transferred into living cells for visualizing the intracellular HClO with a ratiometric time-gated luminescence imaging mode.

As RAW 264.7 cells are known to be able to produce endogenous HClO when they are stimulated with lipopolysaccharide (LPS), interferon-γ (IFN-γ) and 4β-phorbol-12-myristate-13-acetate (PMA),^44^ the feasibility of using the RTLNP for visualizing the endogenous HClO generated in living RAW 264.7 cells was then investigated. After RAW 264.7 cells were pretreated with LPS (100 ng ml^–1^), IFN-γ (50 ng ml^–1^) and PMA (10 nM), the stimulated cells were incubated with the RTLNP (50 μg ml^–1^) for 1.5 h at 37 °C. In this case, obvious quenching of red luminescence and stable green luminescence were observed in the described cells (Fig. 5c). Quantitative analysis of the ratiometric images (Fig. 5c4) showed that the *I*_green_/*I*_red_ value was increased by over 1.81-fold after the LPS/IFN-γ/PMA stimulation. To confirm that the increase in the *I*_green_/*I*_red_ value was indeed induced by the response of the RTLNP to the endogenous HClO, the RAW 264.7 cells were treated with 4-aminobenzoic acid hydrazide (4-ABAH, a MPO inhibitor) during the stimulation.^45^ As expected, both the red and green channels gave bright luminescence images (Fig. 5d), and the increase in the *I*_green_/*I*_red_ value was totally suppressed (Fig. 5d4), which strongly demonstrated the selective luminescence response of the RTLNP to the endogenous HClO generated in the stimulated RAW 264.7 cells.

It is known that macrophage cells respond sensitively to the bacterial infection, in which they produce abundant HClO in the cytoplasm to kill bacteria.^6,46^ So the RTLNP was further employed to detect endogenous HClO in RAW 264.7 cells that were infected by *Escherichia coli* (*E. coli*). After RAW 264.7 cells and *E. coli* were co-incubated for 7 h at 37 °C, the infected cells were sequentially incubated with 50 μg ml^–1^ RTLNP for 1.5 h at 37 °C. As shown in Fig. 5e, the infected RAW 264.7 cells exhibited weak red luminescence and bright green luminescence with a 4.33-fold increase of the *I*_green_/*I*_red_ value (Fig. 5e4) in comparison with the blank control, which further revealed the versatile abilities of the RTLNP for the ratiometric time-gated luminescence imaging of HClO in living cells. It is notable that the above *I*_green_/*I*_red_ value is remarkably larger than that obtained from the LPS/IFN-γ/PMA stimulated RAW 264.7 cells, which suggests that RAW 264.7 macrophage cells are more sensitive to *E. coli* for producing endogenous HClO.

### Application of the RTLNP for ratiometric time-gated luminescence imaging of HClO in animals

**Fig. 6:**
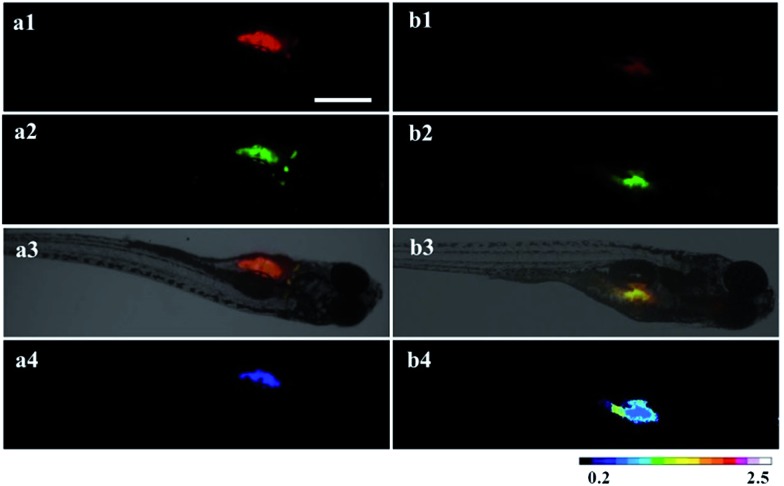
Time-gated luminescence images of the 5-day-old zebrafish loaded with the RTLNP (100 μg ml^–1^) for 1.5 h (a), followed by incubation with HClO (50 μM) for 20 min (b) (1: the images at the red channel; 2: the images at the green channel; 3: overlap of luminescence and bright field images; 4: *I*_green_/*I*_red_ ratiometric images). Scale bar: 500 μm.

To further evaluate the feasibility of using the RTLNP for *in vivo* ratiometric time-gated luminescence bioimaging, at first, zebrafish were chosen as an animal model for studying the uptake of exogenous HClO using the RTLNP as a probe, since zebrafish have been proved to be a highly valuable vertebrate model for *in vivo* imaging in a variety of biological investigations.^47^ After 5-day-old zebrafish were incubated with 100 μg ml^–1^ RTLNP in E3 embryo medium for 1.5 h at 26 °C, the RTLNP-loaded zebrafish were exposed to 50 μM HClO in E3 embryo medium for 20 min at 26 °C, and then their time-gated luminescence images were recorded. As shown in Fig. 6a, the RTLNP particles loaded in the liver, stomach and yolk of the zebrafish exhibited bright luminescence in both the red and green channels, which is similar to the images obtained in the intracellular experiments. In contrast, when the RTLNP-loaded zebrafish were treated with 50 μM HClO for 20 min, a clear change was observed in the luminescence images such that the luminescence signals were obviously decreased in the red channel, while bright luminescence signals were retained in the green channel (Fig. 6b). Quantitative analysis of the ratiometric images showed that the *I*_green_/*I*_red_ value was increased over 1.7-fold after the HClO treatment (Fig. 6a4 and b4).

**Fig. 7:**
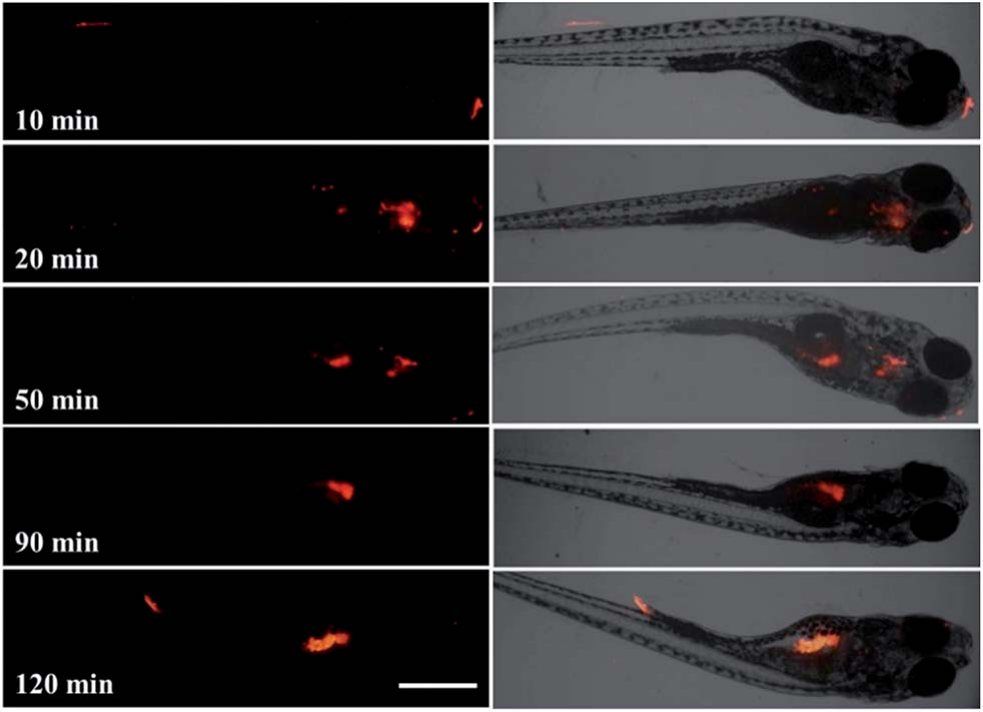
Intracorporal process evaluated by time-gated luminescence imaging at various incubation times (10, 20, 50, 90, and 120 min) after loading zebrafish with the RTLNP (100 μg ml^–1^) (left: the luminescence images of 5-day-old zebrafish treated with the RTLNP at various incubation times; right: overlap of luminescence and bright field images). Scale bar: 500 μm.

For a probe used *in vivo*, it is important to clarify the internalization process of the probe as well as its subsequent behavior in living subjects. Therefore the uptake of RTLNP particles by zebrafish and their intracorporal process were further evaluated by time-gated luminescence imaging at various incubation times. As shown in Fig. 7, after a short incubation time (10 min), some RTLNP particles had adhered to the mouth and tail of the zebrafish. When the incubation time was increased to 20 min, the RTLNP particles were transported into the zebrafish, and mainly accumulated in the head. After 50 min incubation, some of the internalized RTLNP particles moved to the abdominal cavity of the zebrafish, while some RTLNP particles still remained in the head. Upon further increasing the incubation time to 90 min, the RTLNP particles were completely transferred to the abdominal cavity, and localized in the liver, stomach and yolk of the zebrafish. Interestingly, it was observed that the RTLNP particles were excreted in the feces after 120 min incubation. Furthermore, the fate of the RTLNP particles after internalization by the zebrafish was investigated. After the zebrafish were loaded with 100 μg ml^–1^ RTLNP for 1.5 h at 26 °C and carefully washed, the RTLNP-loaded zebrafish were further incubated in a large volume of fresh RTLNP-free medium for 3 h at 26 °C, and then their time-gated luminescence images were recorded. As shown in Fig. S6,† after the above treatment only a few dispersive red luminescence spots were observed in the zebrafish. This result indicates that the RTLNP particles could be cleaned up by the body of the zebrafish, and further suggests that the zebrafish body could control the internal accumulation of the RTLNP to avoid the damage induced by excessive particles. Furthermore, compared to the images obtained using the steady-state imaging mode, the time-gated imaging mode allows the strong autofluorescence of the zebrafish to be substantially suppressed, so that highly specific and background-free images of the zebrafish with clearly long-lived luminescence signals were recorded (Fig. S7†).

**Fig. 8:**
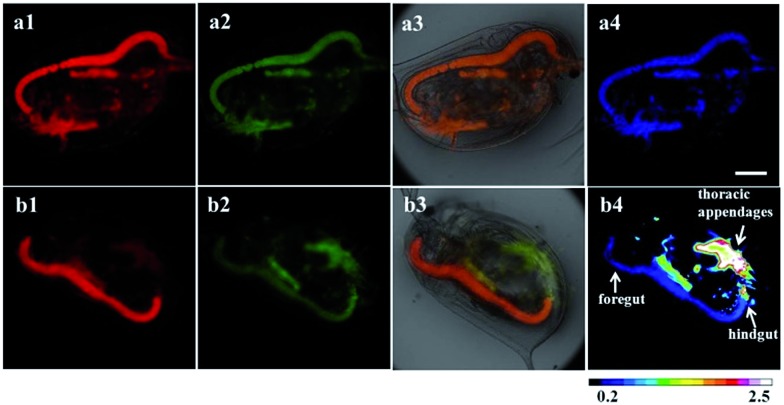
Time-gated luminescence images of *Daphnia magna* treated with the RTLNP (100 μg ml^–1^) for 1.5 h (a), and followed by incubation with HClO (50 μM) for 20 min (b) (1: the images at the red channel; 2: the images at the green channel; 3: overlap of luminescence and bright-field images; 4: *I*_green_/*I*_red_ ratiometric images). Scale bar: 200 μm.

Largely used in our daily lives and various industrial applications as a sanitizer and a bleaching agent,^48–50^ the release of HClO into aquatic environments and its effects on ecotoxicity remain largely unknown. Here, using the RTLNP as a probe we monitored the accumulation of HClO in *Daphnia magna*, a widely used laboratory animal as an indicator of the health of aquatic ecosystems and as a model organism in ecotoxicology,^51,52^ which could provide useful insight for understanding the bioaccumulation and effect of HClO on aquatic organisms. As shown in Fig. 8a, after incubation with 100 μg ml^–1^ RTLNP for 1.5 h at 26 °C, the thoracic appendages and gut of *Daphnia magna* exhibited strong luminescence signals in both the red and green channels. When the RTLNP-loaded *Daphnia magna* were further incubated with 50 μM HClO in culture medium, the red luminescence in the thoracic appendages was remarkably weakened, while the green luminescence in the same location remained stable (Fig. 8b). Quantitative analysis of the ratiometric images showed that the *I*_green_/*I*_red_ value of the RTLNP located in the thoracic appendages was increased over 6.4-fold after incubation with HClO for 20 min (Fig. 8a4 and b4). In contrast, the *I*_green_/*I*_red_ value of the RTLNP located in the gut increased slowly and very little. The local *I*_green_/*I*_red_ values in the foregut and hindgut were only 0.23-fold and 1.8-fold increased, respectively (Fig. 8b4). This phenomenon might be ascribed to the convenient contact of the RTLNP with HClO in thoracic appendages and the relatively high concentration of naturally existing bio-antioxidants in the esophagus and midgut.

By taking advantage of the ability of the RTLNP to respond to HClO quickly, the location and accumulation of HClO in *Daphnia magna* were dynamically monitored by sequential luminescence imaging in a real-time manner. After the RTLNP-loaded *Daphnia magna* were exposed to 200 μM HClO in culture medium at 26 °C, the luminescence of the RTLNP in *Daphnia magna* was recorded in real-time using the sequential time-gated luminescence imaging mode and a movie was made from a sequence of the images. As shown in Fig. S8 and the video (in the ESI†), the luminescence of the RTLNP particles located in the thoracic appendages of *Daphnia magna* changed from red to green, and the local *I*_green_/*I*_red_ value was increased over 3.6-fold within 30 seconds incubation (Fig. S9†), which implies that the red luminescence of BHHBB–Eu^3+^ on the surface of the RTLNP was rapidly and substantially quenched by HClO, and the green luminescence of PTTA–Tb^3+^ in the core of the RTLNP was not affected by HClO. In contrast, the luminescence of the RTLNP located in the gut of *Daphnia magna* remained bright red and the local *I*_green_/*I*_red_ value was almost unchanged within 30 seconds incubation (Fig. S9†), owing to the low concentration of HClO in the gut. Furthermore, as shown in Fig. S9,† a little more HClO molecules were accumulated in the hindgut of *Daphnia magna* than in the foregut. All of the above results demonstrated the practical applicability of the RTLNP as a probe for the real-time monitoring of HClO in complex living bodies.

## Conclusion

We have developed a novel dual-emissive nanoprobe through decorating the surface of terbium-complex-encapsulated silica nanoparticles with a β-diketonate–Eu^3+^ complex for *in vivo* ratiometric time-gated imaging of HClO. The design of this RTLNP is based on our new finding that the strong long-lived luminescence of the β-diketonate–Eu^3+^ complex can be rapidly, selectively and sensitively quenched by HClO. The as-prepared RTLNP probe displays an ideal single-excitation, dual-emission property with well-resolved luminescence peaks, and possesses both the advantages of ratiometric and time-gated detection modes to exhibit high accuracy and sensitivity. The potential of the RTLNP has been comprehensively evaluated by imaging exogenous and endogenous HClO in cancerous cells, mapping of HClO in zebrafish, and monitoring of HClO uptake in *Daphnia magna*, which could be anticipated to be a useful tool for visualizing HClO *in vivo* to facilitate understanding of the roles of HClO in physiology, disease states and ecotoxicology. Importantly, such a design strategy provides a facile approach for constructing a new ratiometric time-gated luminescence nanoprobe for the detection of active biomolecules in living organisms with high specificity and contrast.

## Experimental

### Materials and physical measurements

The ligands PTTA, BHHBB, chlorosulfonated-BHHBB (BHHBCB), and the complex BHHBB–Eu^3+^ were synthesized according to the literature methods.^35,41^ Triton X-100, APTES, TEOS, *N*-hydroxysuccinimide (NHS), and 1-ethyl-3-(3-dimethylamino-propyl) carbodiimide hydrochloride (EDC) were purchased from Acros Organic. Cultured RAW 264.7 macrophage cells and *E. coli* were obtained from Dalian Medical University. Cultured *Daphnia magna* and zebrafish were obtained from Professor Jingwen Chen's group at the School of Environmental Science and Technology, Dalian University of Technology. Unless otherwise stated, all chemical materials were purchased from commercial sources and used without further purification.

^1^H NMR spectra were measured on a Bruker Avance spectrometer (400 MHz). Mass spectra were measured on a LTQ Orbitrap XL mass spectrometer. Time-gated luminescence spectra were measured on a Perkin-Elmer LS 50B luminescence spectrometer with the settings of delay time, 0.2 ms; gate time, 0.4 ms; cycle time, 20 ms; excitation slit, 5 nm; and emission slit, 5 nm. Luminescence lifetimes were measured on a FS5 spectrofluorometer from Edinburgh Instruments. All bright-field and luminescence imaging measurements were carried out on a laboratory-use true color time-gated luminescence microscope.^43^

### Reaction of BHHBB–Eu^3+^ with HClO

To 8.0 ml of acetonitrile–0.05 M PBS buffer (1 : 1, v/v) of pH 7.4 containing 1.0 μmol HClO was added 2.0 ml acetonitrile solution containing 0.05 μmol BHHBB–Eu^3+^ dropwise. The mixture was agitated for 30 min and the reaction was monitored by fluorometry to check the complete oxidation of BHHBB–Eu^3+^. The solution was neutralized by oxalic acid and lyophilized. The residue was purified by silica gel column chromatography eluting with 10% water in acetonitrile and washed with tiny amounts of acetone to afford the product as a white powder (28 mg, 80.9% yield). ^1^H NMR (500 MHz, d_6_-DMSO): *δ* = 4.01 (s, 4H, CH_2_), 7.15–7.22 (m, 8H, Ar), 7.83 (d, *J*(H,H) = 8.0 Hz, 4H, Ar). ^13^C NMR (125 MHz, d_6_-DMSO): *δ* = 38.0 (CH_2_), 126.7 (Ar), 128.6 (Ar), 128.7 (Ar), 129.4 (Ar), 130.4 (Ar), 138.3 (Ar), 145.5 (Ar), 167.2 (C=O). ESI-HR-MS (*m*/*z*): 345.1132 ([M–H]^–^, 100%, calcd 345.1122).

### Synthesis of APTES–BHHBB–Eu^3+^ conjugate

BHHBCB (2.0 μmol) and APTES (4.0 μmol) were dissolved in acetone (50 μl), and the mixture was stirred at 37 °C for 6 h. Then BHHBB (2.0 μM) was added, and the mixture was stirred for another 30 min. After that, a water solution of EuCl_3_ (100 μl, 50 mM) was added with stirring, and the reaction was allowed to continue for another 2 h. Finally 50 μl of DMSO was added to the mixture to make the reaction solution homogeneous. The conjugate was directly used in the next covalent decoration procedure for preparing the RTLNP without further purification.

### Preparation of nanoparticles

PTTA–Tb^3+^-encapsulated silica nanoparticles were prepared according to our previous method.^38,39^ The prepared PTTA–Tb^3+^-encapsulated silica particles were dispersed in 40 ml anhydrous ethanol and ultrasonicated for 30 min to afford a homogeneous solution. Then 60 μl of concentrated aqueous ammonia (25%) was added into the solution, followed by dropwise addition of the APTES–BHHBB–Eu^3+^ conjugate stock solution. The reaction was allowed to continue for 24 h. The desired RTLNP nanoparticles were isolated by centrifuging and washing with ethanol and water several times to remove the surfactant and unreacted materials.

### Reaction of the RTLNP with HClO

The reaction of the RTLNP with HClO was performed in 1 : 4 ethanol–0.05 M PBS buffer of pH 7.4. After the solution of the RTLNP (50 μg ml^–1^) was incubated with different concentrations of HClO for 10 min, the excitation and emission spectra of the solutions were measured using the time-gated mode.

### Reactions of the RTLNP with ROS and RNS

All the reactions were carried out in 1 : 4 ethanol–0.05 M PBS buffer of pH 7.4 (except for ^1^O_2_) with the same concentration of the RTLNP (50 μg ml^–1^) for 0.5 h at room temperature. Then the luminescence intensities of the solutions were measured using the time-gated mode. The stock solution of ClO^–^ was prepared by diluting a commercial NaClO solution, and the concentration of ClO^–^ was determined based on the molar extinction coefficient of ClO^–^ at 292 nm (350 M^–1^ cm^–1^). Hydrogen peroxide solution was prepared by diluting a commercial 30% H_2_O_2_ solution, and the concentration of H_2_O_2_ was determined based on the molar extinction coefficient of H_2_O_2_ at 240 nm (43.6 M^–1^ cm^–1^). Hydroxyl radical (˙OH) was generated in the Fenton system from ferrous ammonium sulfate and hydrogen peroxide.^53^ A superoxide anion radical (O_2_^–^˙) was generated from the xanthine–xanthine oxidase system.^54^ Peroxynitrite (ONOO^–^) was generated using 3-morpholinosydnonimine (SIN-1) as a donor.^55^ Nitric oxide (NO) was generated using 1-hydroxy-2-oxo-3-(3-amino-propyl)-3-methyl-1-triazene (NOC-13) as a donor.^56^ Singlet oxygen was chemically generated from the MoO_4_^2–^–H_2_O_2_ system in alkaline medium, and incubated with the RTLNP in a 1 : 4 ethanol–0.05 M sodium carbonate buffer of pH 10.5.

### MTT assay

The cytotoxicity of the RTLNP to RAW 264.7 cells was measured by the MTT assay using the previously reported method.^42^ RAW 264.7 cells cultured in DMEM medium, containing 10% FBS (fetal bovine serum) and antibiotics (100 units per ml penicillin and 100 μg ml^–1^ streptomycin), were washed with an isotonic saline solution (140 mM NaCl, 10 mM glucose and 3.5 mM KCl), and then incubated with different concentrations of the RTLNP (0, 30, 50, 70, 100 and 120 μg ml^–1^) for 24 h at 37 °C in a 5% CO_2_/95% air incubator. After the culture medium was removed, the cells were further incubated with the PBS buffer containing 0.5 mg ml^–1^ of MTT for 4 h in the incubator. After the supernatants were removed, the cells were dissolved in DMSO, and then the absorbance at 490 nm was measured.

### Luminescence imaging of exogenous HClO in living cells

RAW 264.7 cells, cultured on a 35 mm glass-bottom culture dish (*φ* 20 mm) in DMEM containing 10% FBS and antibiotics (100 units per ml penicillin and 100 μg ml^–1^ streptomycin) in a humidified atmosphere of 95% air and 5% CO_2_ at 37 °C, were washed three times with an isotonic saline solution, and then incubated in an isotonic saline solution containing 50 μg ml^–1^ RTLNP and 1.0 mg ml^–1^ solubilizer, cremophor C040, for 1.5 h at 37 °C in a 5% CO_2_/95% air incubator. The RTLNP-loaded RAW 264.7 cells were washed three times with PBS buffer (137 mM NaCl, 2.7 mM KCl, 10.1 mM Na_2_HPO_4_ and 1.8 mM KH_2_PO_4_, pH 7.4), and then incubated in the PBS buffer containing 50 μM HClO for 10 min under the same conditions. The cells were washed three times with the PBS buffer, and then subjected to the time-gated luminescence imaging measurements on the microscope (excitation filter, 330–380 nm; dichroic mirror, 400 nm; emission filters, >590 nm LP filter for red luminescence, and 540 ± 25 nm BP filter for green luminescence). The time-gated luminescence imaging measurements were carried out with the conditions of delay time, 33 μs; gate time, 1.0 ms; lamp pulse width, 80 μs; and exposure time, 200 ms.

### Luminescence imaging of endogenous HClO in living cells

For the imaging of LPS/IFN-γ/PMA-induced intracellular HClO generation in RAW 264.7 cells, the cells were incubated with culture media containing LPS (100 ng ml^–1^) for 16 h, followed by 4 h incubation with 50 ng ml^–1^ IFN-γ and 30 min incubation with 10 nM PMA. The stimulated cells were incubated in the isotonic saline solution containing 50 μg ml^–1^ RTLNP and 1.0 mg ml^–1^ solubilizer, cremophor C040, for 1.5 h at 37 °C in a 5% CO_2_/95% air incubator, and then subjected to the time-gated luminescence imaging measurements on the microscope. For the control experiment, the LPS-IFN-γ-stimulated cells were incubated with PMA and 4-ABAH (50 μM) for 2 h. After that, the cells were loaded with 50 μg ml^–1^ RTLNP, rinsed three times with an isotonic saline solution, and then their luminescence images were recorded.

For the imaging of bacteria-induced HClO generation in RAW 264.7 cells, the cells were pretreated with *E. coli* (2 × 10^5^ CFU ml^–1^) under cell culture conditions for 7 h, and then washed three times with the isotonic saline solution. The stimulated cells were treated with 50 μg ml^–1^ RTLNP for 1.5 h, rinsed three times with isotonic saline solution, and then their luminescence images were recorded.

### Luminescence imaging of HClO in zebrafish

All stages of zebrafish were maintained in E3 embryo medium without methylene blue (15 mM NaCl, 0.5 mM KCl, 1.0 mM MgSO_4_, 1.0 mM CaCl_2_, 0.15 mM KH_2_PO_4_, 0.05 mM Na_2_HPO_4_, 0.7 mM NaHCO_3_; pH 7.5) at 26 °C with a 12 h light/12 h dark cycle. The zebrafish were incubated with an E3 embryo medium containing 100 μg ml^–1^ RTLNP for different times at 26 °C. After washing three times with the culture medium, the RTLNP-loaded zebrafish were subjected to the time-gated luminescence imaging measurements, or further treated with 50 μM HClO for 20 min. The HClO-treated zebrafish were washed three times with the E3 embryo medium before use for luminescence imaging.

### Luminescence imaging of HClO in *Daphnia magna*

*Daphnia magna* were cultured in nonchlorinated tap water that was aerated for 3 days and saturated in dissolved oxygen at 20 °C under a cool-white fluorescent light with a 14 : 10 h light : dark photoperiod. The culture medium was renewed three times a week. *Scenedesmus obliquus* were fed to *Daphnia magna* daily. The newborn *Daphnia magna* (age < 48 h) were first loaded with 100 μg ml^–1^ RTLNP for 1.5 h at 26 °C. After washing three times with the culture medium, the RTLNP-loaded *Daphnia magna* were incubated with 50 μM HClO in PBS buffer (pH 7.4) at 26 °C, and their time-gated luminescence images were recorded at different incubation times.
