# Supplementary material for: Dual-emissive nanoarchitecture of lanthanide-complex-modified silica particles for in vivo ratiometric time-gated luminescence imaging of hypochlorous acid
Source: Chem Sci. 2016 Jul 29;8(1):150–9. doi: 10.1039/c6sc02243j (PMC5308275; doi:10.1039/c6sc02243j)
Supplement: Supplementary file 1 [file SC-008-C6SC02243J-s001.pdf]

Electronic Supplementary Material (ESI) for Chemical Science.
This journal is © The Royal Society of Chemistry 2016

Supporting information for

Dual-Emissive Nanoarchitecture of Lanthanide Complex-Modified Silica Particles for in vivo Ratiometric Time-Gated Luminescence Imaging of Hypochlorous Acid

Hua Ma, Bo Song,* Yuanxiu Wang, Deyuan Cong, Yufei Jiang and Jingli Yuan*

State Key Laboratory of Fine Chemicals, School of Chemistry, Dalian University of Technology, Dalian 116024, P. R. China

*Corresponding authors. Tel/Fax: +86-411-84986041

E-mail addresses: bo.song@dlut.edu.cn (B. Song); jlyuan@dlut.edu.cn (J. Yuan).

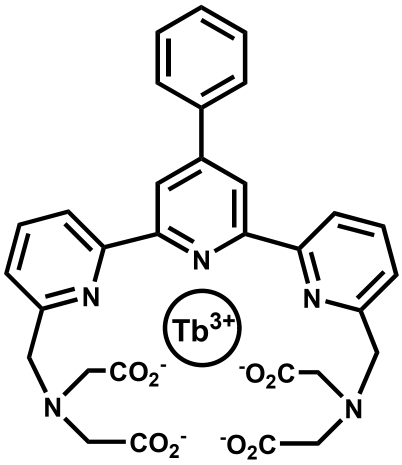

Fig. S1. Chemical structure of PTTA-Tb^{3+} chelate.

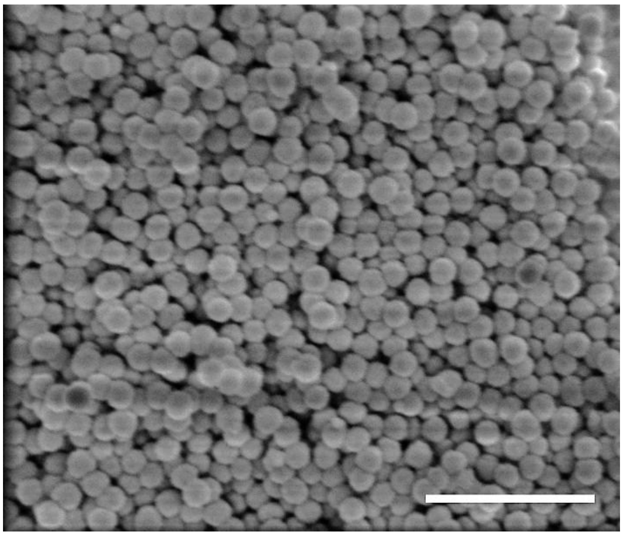

Fig. S2. Scanning electronic microscopy (SEM) image of RTLNP particles. Scale bar: 500 nm.

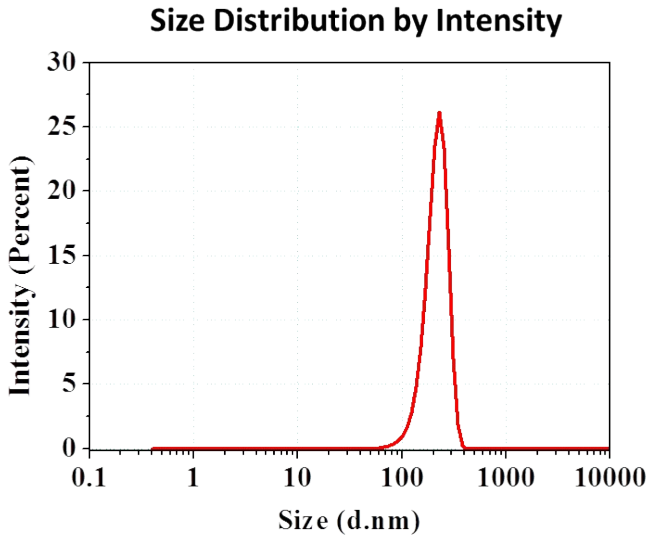

Fig. S3. The hydrated particle size distribution of RTLNP particles in water by dynamic light scattering (DLS) measurement (DLS diameter: 241.4 nm).

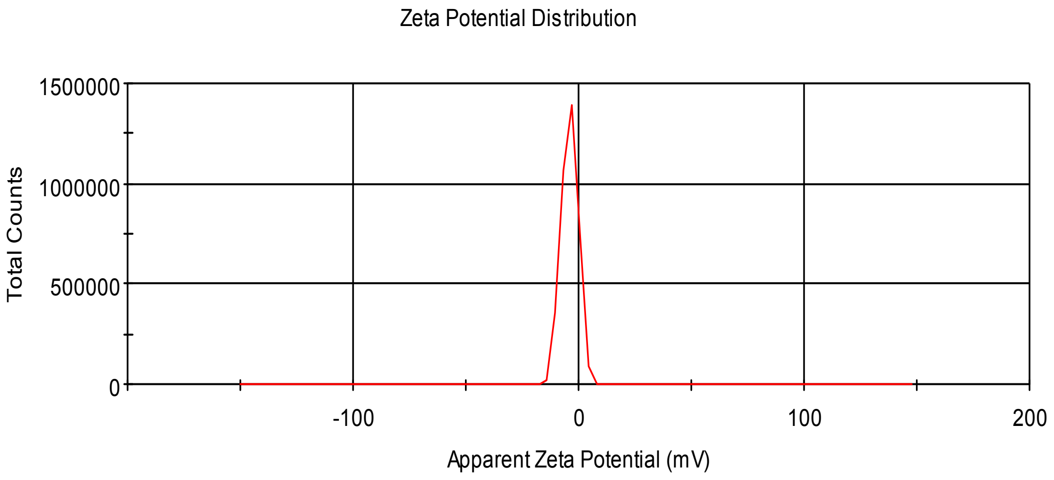

Fig. S4. The zeta potential of RTLNP particles in 0.01 M PBS buffer of pH 7.4, containing 1.0 mg ml^{-1} solubilizer, cremophor C040 (-3.72 mV).

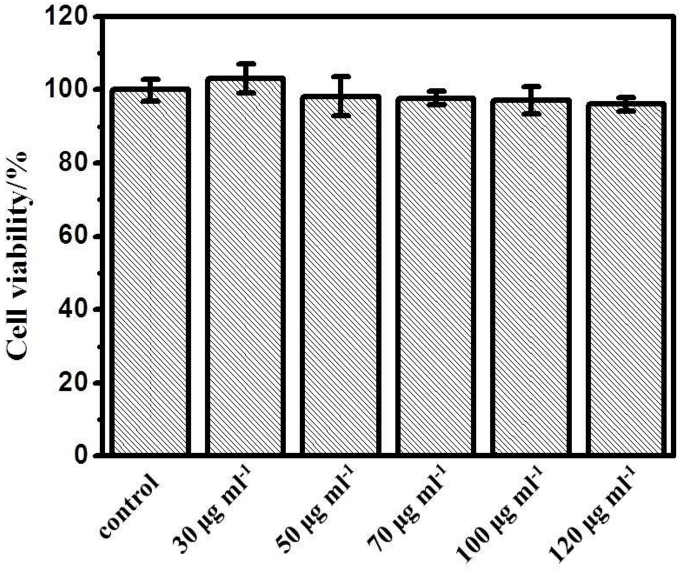

Fig. S5. Viabilities of Raw 264.7 cells after incubated with different concentrations of RTLNP for 24 h.

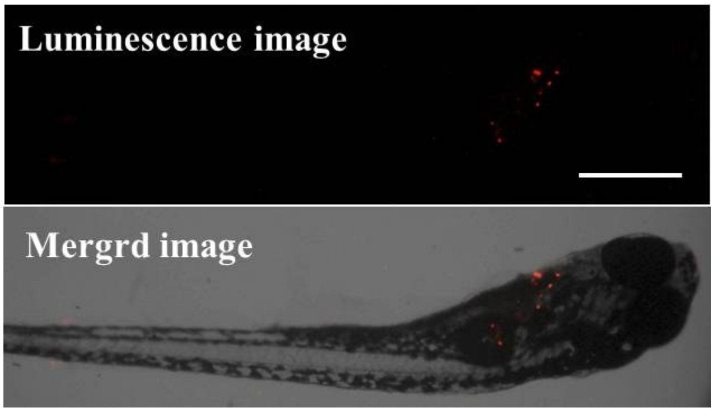

Fig. S6. Luminescence images of 5-day-old zebrafish loaded with RTLNP (100 μg ml^{-1}) for 1.5 h, followed by incubation with fresh culture medium for 3 h. Scale bar: 500 μm.

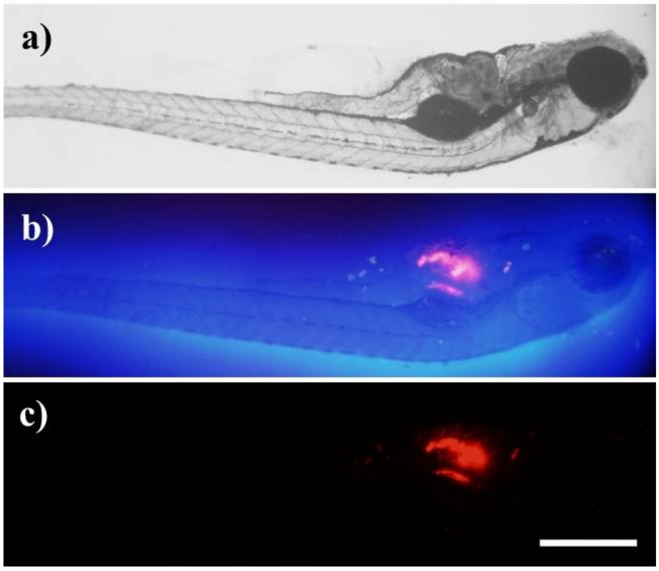

Fig. S7. Bright-field, steady-state luminescence and time-gated luminescence images of 5-day-old zebrafish loaded with RTLNP (100 μg ml^{-1}) for 1.5 h (no emission filter was used for the imaging). Scale bar: 500 μm.

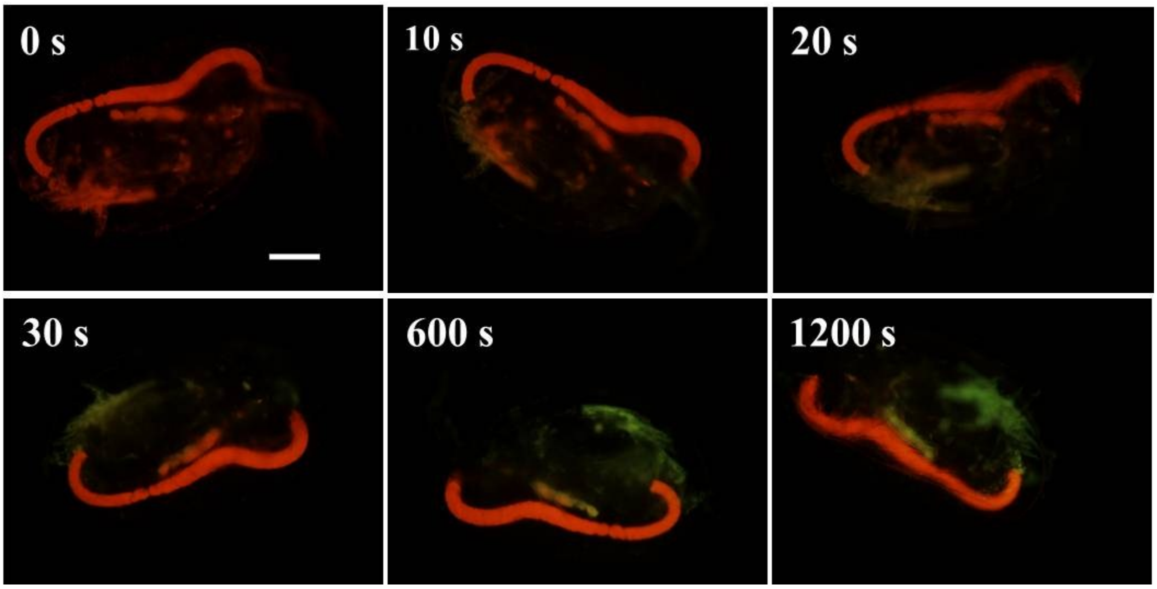

Fig. S8. Time-gated luminescence images of *Daphnia magna* loaded with 100 μg ml^{-1} RTLNP for 1.5 h, and followed by incubation with 50 μM HClO at different incubation times (no emission filter was used for the imaging).

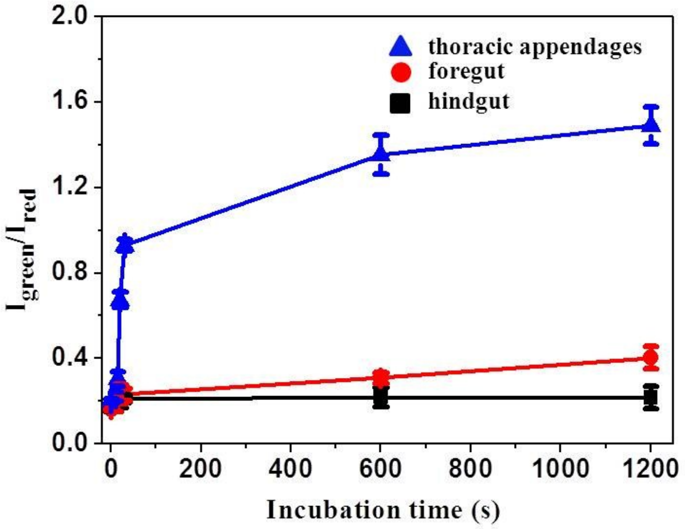

Fig. S9. Time-dependent accumulations of HClO in thoracic appendages, foregut and hindgut of *Daphnia magna*. *Daphnia magna* were loaded with 100 μg ml^{-1} RTLNP for 1.5 h, and followed by incubation with 50 μM HClO at different incubation times. The accumulations of HClO in different organs of *Daphnia magna* were evaluated by the local luminescence ratio (I_{green}/I_{red}) of RTLNP.

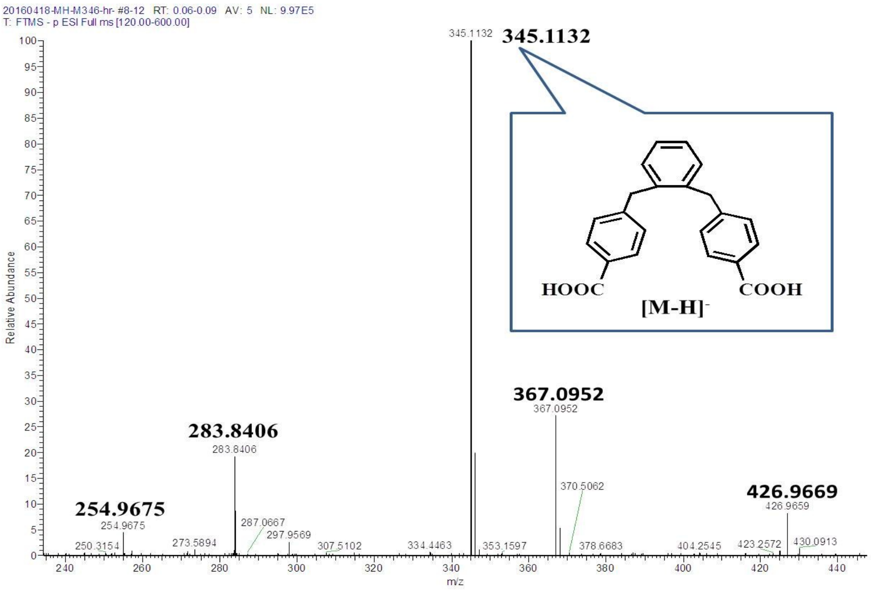

Fig. S10. HRMS of the oxidation product of BHHBB-Eu^{3+} by HClO.

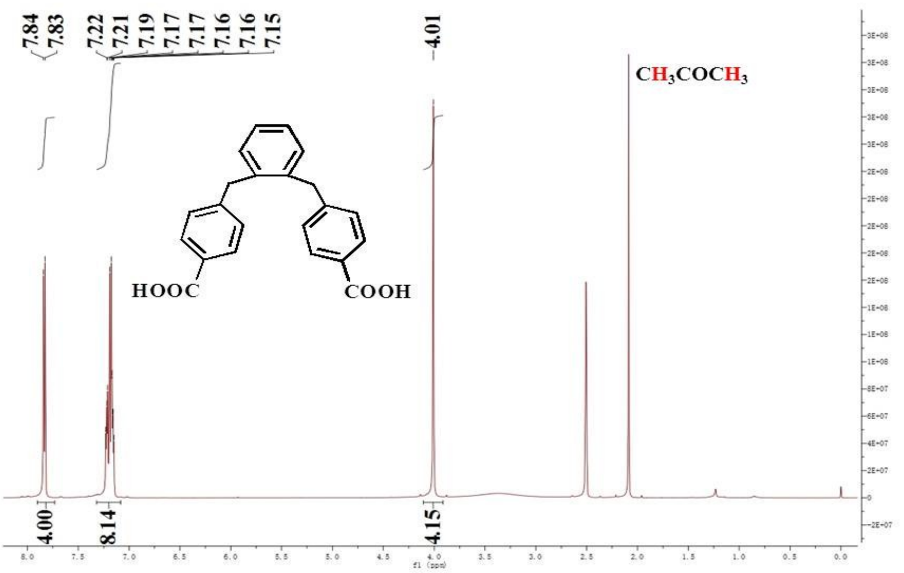

Fig. S11. ^{1}H NMR spectrum (500 MHz, in d_6-DMSO) of the oxidation product of BHHBB-Eu^{3+} by HClO.

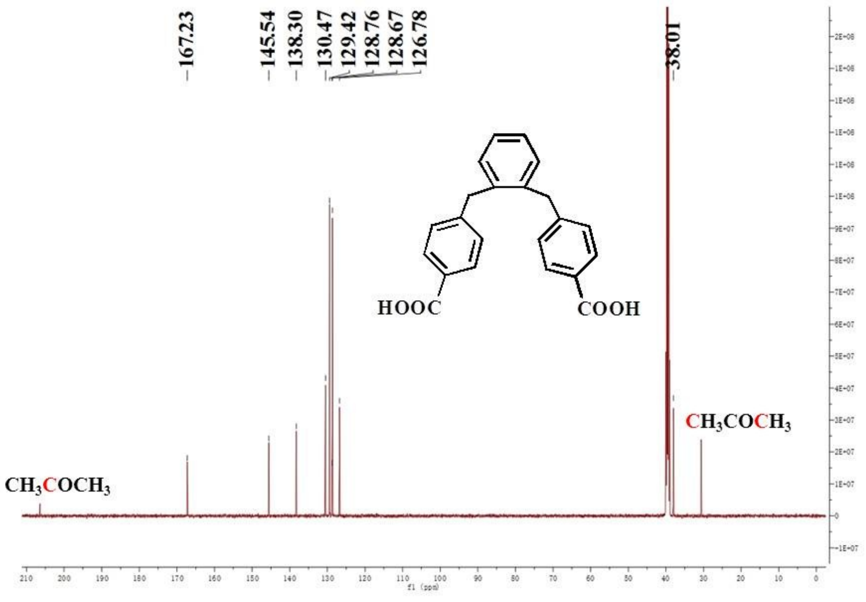

Fig. S12. ^{13}C NMR spectrum (125 MHz, in d_6-DMSO) of the oxidation product of BHHBB-Eu^{3+} by HClO.
